# Distinctive Growth and Transcriptional Changes of the Diatom *Seminavis robusta* in Response to Quorum Sensing Related Compounds

**DOI:** 10.3389/fmicb.2020.01240

**Published:** 2020-06-09

**Authors:** Frederike Stock, Gust Bilcke, Sam De Decker, Cristina Maria Osuna-Cruz, Koen Van den Berge, Emmelien Vancaester, Lieven De Veylder, Klaas Vandepoele, Sven Mangelinckx, Wim Vyverman

**Affiliations:** ^1^Research group Protistology and Aquatic Ecology, Department of Biology, Faculty of Sciences, Ghent University, Ghent, Belgium; ^2^Department of Applied Mathematics, Computer Science and Statistics, Faculty of Sciences, Ghent University, Ghent, Belgium; ^3^Department of Plant Biotechnology and Bioinformatics, Ghent University, Ghent, Belgium; ^4^VIB Center for Plant Systems Biology, Ghent, Belgium; ^5^Bioinformatics Institute Ghent, Ghent University, Ghent, Belgium; ^6^Research group Synthesis, Bioresources and Bioorganic Chemistry (SynBioC), Department of Green Chemistry and Technology, Ghent University, Ghent, Belgium

**Keywords:** *N*-acyl homoserine lactones, AHLs, tetramic acid, quorum sensing, diatoms, *Seminavis robusta*, RNA-seq, interkingdom-signaling

## Abstract

In aquatic habitats, diatoms are frequently found in association with Proteobacteria, many members of which employ cell-to-cell communication via *N*-acyl homoserine lactones (AHLs). It has been suggested that diatoms could distinguish between beneficial and algicidal bacteria in their surroundings by sensing AHLs. Although some microalgae can interfere with AHL signaling, e.g., by releasing AHL mimics or degrading them, molecular responses to AHLs in microalgae are still unclear. Therefore, we tested the effects of short-chained AHLs, i.e., *N*-hexanoyl homoserine lactone (C6-HSL), *N*-3-hydroxyhexanoyl homoserine lactone (OH-C6-HSL), and *N*-3-oxohexanoyl homoserine lactone (oxo-C6-HSL) and long-chained AHLs, i.e., *N*-tetradecanoyl homoserine lactone (C14-HSL), *N*-3-hydroxytetradecanoyl homoserine lactone (OH-C14-HSL), and *N*-3-oxotetradecanoyl homoserine lactone (oxo-C14-HSL), on growth of the benthic diatom *Seminavis robusta*. All tested short-chained AHLs did not affect diatom growth, while long-chained AHLs promoted (C14-HSL) or inhibited (OH-C14-HSL and oxo-C14-HSL) growth. To investigate the physiological effects of these long-chained AHLs in more detail, an RNA-seq experiment was performed during which *S. robusta* was treated with the growth-promoting C14-HSL and the growth-inhibiting oxo-C14-HSL. One tetramic acid was also tested (TA14), a structural rearrangement product of oxo-C14-HSL, which also induced growth inhibition in *S. robusta*. After 3 days of treatment, analysis revealed that 3,410 genes were differentially expressed in response to at least one of the compounds. In the treatment with the growth-promoting C14-HSL many genes involved in intracellular signaling were upregulated. On the other hand, exposure to growth-inhibiting oxo-C14-HSL and TA14 triggered a switch in lipid metabolism towards increased fatty acid degradation. In addition, oxo-C14-HSL led to downregulation of cell cycle genes, which is in agreement with the stagnation of cell growth in this treatment. Combined, our results indicate that bacterial signaling molecules with high structural similarity induce contrasting physiological responses in *S. robusta*.

## Introduction

Diatoms have co-occurred with marine bacteria for millions of years. A suite of diatom-bacteria interactions have been described, ranging from pathogenic bacteria inducing diatom cell lysis to mutualistic relationships (Amin et al., 2012; Meyer et al., 2017; Seymour et al., 2017). While the majority of studies so far focused on planktonic diatoms, biofilm-inhabiting diatoms co-exist with dense bacterial populations in a polymeric matrix, which facilitates close interactions between them. The majority of diatoms’ satellite bacteria, isolated from field and laboratory cultures, belong to the phyla Proteobacteria and Bacteroidetes (Schäfer et al., 2002; Grossart et al., 2005; Amin et al., 2012; Buchan et al., 2014; Green et al., 2015). Many Proteobacteria engage in a cell-to-cell signaling process called quorum sensing (QS), which allows them to coordinate their behavior at the population level (Waters and Bassler, 2005). The most extensively studied class of bacterial QS molecules are *N*-acyl homoserine lactones (AHLs). These molecules consist of a lactone ring coupled to an acyl chain, which can vary in length from 4 up to 18 carbon atoms (Hmelo, 2017; Waters and Bassler, 2005). In addition, AHLs can have a substitution at the C_3_ position of the acyl chain in the form of a hydroxyl- or keto-group (Waters and Bassler, 2005), further contributing to the functional diversity of these compounds in bacterial signaling.

Interestingly, AHL-based signaling is not only limited to bacteria. For example, AHL-producing bacteria affect the swimming speed of zoospores of the seaweed *Ulva* via modulation of calcium signaling (Wheeler et al., 2006; Joint et al., 2007). Meanwhile, the green microalga *Chlamydomonas rheinhardtii* produces more than a dozen compounds that mimic AHLs, which can interfere with bacterial QS circuits (Teplitski et al., 2004). Similarly, an AHL inactivation mechanism was identified in the diatom *Nitzschia* cf *pellucida*, which uses a haloperoxidase to halogenate 3-oxo-AHLs, resulting in the cleavage of the *N*-acyl chain and deactivation of their signaling potential (Syrpas et al., 2014).

In this study, we investigated the effect of AHLs on the growth and gene expression patterns of the marine benthic diatom *Seminavis robusta*. In previous studies, several associated bacteria were isolated from xenic *S. robusta* cultures, including Gammaproteobacteria (e.g., *Marinobacter* sp.) as well as Alphaproteobacteria (e.g., *Roseovarius* sp.) and Bacteroidetes (e.g., *Maribacter* sp.) (Cirri et al., 2018; De Troch et al., 2010). Laboratory experiments indicated that the bacterial community associated with *S. robusta* can affect the sexual reproduction success of diatoms, with *Maribacter* sp. and *Marinobacter* sp. reducing reproductive output and *Roseovarius* sp. enhancing it (Cirri et al., 2018). Considering that *Marinobacter* (Gram et al., 2002) as well as *Roseovarius* (Wagner-Dobler et al., 2005) produce short- and long-chained AHLs, as do many other marine members of the Alpha- and Gammaproteobacteria (Wagner-Dobler et al., 2005; Girard, 2019), this prompted us to explore the effects of AHLs on *S. robusta*. Specifically, we assessed (a) growth effects of structurally similar short- and long-chained AHLs (i.e., C6-HSL, OH-C6-HSL, oxo-C6-HSL, C14-HSL, OH-C14-HSL, and oxo-C14-HSL), and (b) effects on gene expression of selected long-chained AHLs, i.e., C14-HSL, oxo-C14-HSL, and its tetramic acid rearrangement product, TA14. The latter compound was included as tetramic acids inhibit photosynthetic electron flow (Stock F. et al., 2019). Our results show that short-chained AHLs did not induce any growth response in *S. robusta*, whereas long-chained AHLs caused significant but contrasting effects. Specifically, C14-HSL stimulated diatom growth whereas OH-C14-HSL, oxo-C14-HSL, and TA14 inhibited growth, highlighting that structurally similar compounds induce diverse physiological effects. Subsequent RNA-seq analysis, supported by the availability of genomic resources (Osuna-Cruz et al., 2020), showed that treatment with all tested compounds (C14-HSL, oxo-C14-HSL, and TA14) led to differential expression of genes related to signaling and oxidative stress. Moreover, oxo-C14-HSL and TA14 treatment affected fatty acid metabolism and photosynthesis, which were unaffected by C14-HSL. These metabolic changes could explain why treatment with oxo-C14-HSL and TA14 inhibited diatom growth. Interestingly, cell cycle genes were strongly downregulated in the treatment with oxo-C14-HSL, which could underlie the observed cell cycle arrest. Taken together, our findings suggest that *S. robusta* can not only sense AHLs and TA in its surroundings but is also able to distinguish between signaling compounds with high structural similarity.

## Materials and Methods

### Organism and Culture Conditions

*Seminavis robusta* 85A (mating type MT+, accession number DCG 0105) was obtained from the Belgian Coordinated Collections of Microorganisms^1^ (BCCM/DCG). Diatoms were cultured in artificial seawater (=ASW: 34.5 g L^–1^ Tropic Marin^®^, 0.08 g L^–1^ HCO_3_^–^) supplemented with Guillard’s F2 (Sigma-Aldrich) in a 12 h:12 h dark/light regime at 18°C. The light intensity was approximately 25 μmol photons m^–2^ s^–1^. Prior to the experiments, diatom cultures were made axenic by treating them with an antibiotic mix (500 μg mL^–1^ penicillin, 500 μg mL^–1^ ampicillin, 100 μg mL^–1^ streptomycin, and 50 μg mL^–1^ gentamicin) for a week and replacing the antibiotic supplemented medium every second day. During the experiments, *S. robusta* was cultured in artificial seawater containing Guillard’s F2 without antibiotics.

### Compound Preparation

C6-HSL, OH-C6-HSL, oxo-C6-HSL, C14-HSL, OH-C14-HSL, oxo-C14-HSL, and TA14 were synthesized using standard procedures (Cao et al., 1995; Kaufmann et al., 2005; Hodgkinson et al., 2011). Briefly, C6-HSL, oxo-C6-HSL, C14-HSL, and oxo-C14-HSL were synthesized according to the method described in Hodgkinson et al. (2011) and syntheses of OH-C6-HSL and OH-C14-HSL were performed as described in Cao et al. (1995). TA14 was synthesized as reported in Kaufmann et al. (2005). After purification via column chromatography on silica gel (ethyl acetate:petroleum ether 3:2 to 4:1) (for AHLs) or recrystallization from hexane (for TA14), the purity of all compounds was >95% as determined by reversed phase HPLC analysis (integration at 220 nm). For growth- and RNA-seq experiments, working solutions were prepared for each compound in DMSO such that the final concentration of DMSO in each culture was always 0.5% (*v/v*). For the growth experiments, C6-HSL, OH-C6-HSL, oxo-C6-HSL, C14-HSL, OH-C14-HSL, and oxo-C14-HSL were added at final concentrations of 20, 50, and 100 μM each. For the RNA-seq experiment, the final compound concentration in each treatment was 40 μM for C14-HSL and oxo-C14-HSL and 5 μM in the TA14 treatment. A lower concentration of TA14 was chosen due to its strong algicidal properties resulting in cell death at 40 μM (Stock F. et al., 2019), while at a concentration of 5 μM a growth arrest is induced while cells remain viable.

### Cell Culturing

For growth experiments, cells were cultured in 48-well plates (Greiner Cellstar, Sigma-Aldrich), treated with DMSO (control) or with DMSO and either C6-HSL, OH-C6-HSL, oxo-C6-HSL, C14-HSL, OH-C14-HSL, and oxo-C14-HSL. Three replicates were used for each treatment. Cell proliferation was monitored daily for one week using a PAM-fluorometer (MAXI Imaging PAM M-series, Walz Mess-und Regeltechnik). The PAM-fluorometer recorded minimum fluorescence, *F*_0_, which served as a proxy for biomass (Stock W. et al., 2019).

To begin the RNA-seq experiment, cells grown in different cell culture flasks (175 cm^2^ Greiner Cellstar, Sigma-Aldrich) were adjusted to the same *F*_0_ using a PAM-fluorometer and each culture flask was filled with 150 mL of cell suspension. In parallel, a 48-well plate was inoculated with a cell suspension of the same density as used in the cell culture flasks. The wells were filled with a volume of 857 μL to obtain an identical volume:surface ratio as the cell culture flasks. C14-HSL, oxo-C14-HSL, and TA14 were added simultaneously to flasks and the 48-well plate at the same final concentrations. Cell growth in flasks was determined by measuring *F*_0_ every day for 3 days. Cell growth in the 48-well plates was determined by taking high resolution pictures of a defined area (3 × 3 pictures, total area: 2,200 × 1,590 μm) in the center of each well with a Cytation 5 Cell Imaging multimode reader (Biotek) every day. The number of cells in the photographed area was counted using Gen5 Software (Biotek) in bright field mode. Cell counts for the photographed surface area were then extrapolated to calculate the cell density for the entire 1 cm^2^ well.

### Statistical Analysis of Cell Counts

Statistical analysis of cell counts was performed using a quasi-Poisson generalized linear model (GLM) with log link. The model included a treatment effect (consisting of a control and three treatment levels: C14-HSL, oxo-C14-HSL, and TA14), a day effect (day 0 to day 3) and their interaction effect. Statistical tests, assessing differences in average cell count between each treatment and the control on days 1 to 3, were performed using Wald tests by constructing linear combinations of the model parameters. We account for the multiplicity of tests on a global 5% significance level (Bretz et al., 2011).

### Cell Harvesting

After 72 h of treatment, cells were removed from the culture flasks using a cell scraper and homogenized by shaking. Subsequently, cells were collected on a Versapor filter (3 μm pore size, 25 mm diameter, PALL). For each treatment, cells from five flasks were collected on five separate filters resulting in five replicates. After harvesting, the filters with the cells were transferred into Eppendorf tubes and immediately placed in liquid nitrogen. Prior to RNA extraction, filters were stored at −80°C.

### RNA Extraction

RNA was extracted using the RNeasy Mini kit (QIAGEN) following the manufacturer’s instructions with some modifications. Briefly, 1 mL RTL buffer was added to the tube and cells were scraped from the filter. The filter was removed and silicon carbide beads (1 mm, BioSpec) were added. Cells were lysed by beating them for 3 × 1 min on a beating mill (Retsch) at a frequency of 20 Hz. The lysate was then transferred to a QIAshredder spin column and centrifuged twice for 2 min at full speed. For the rest of the protocol the manufacturer’s instructions were followed including the recommended DNA digestion.

### Genome Annotation of *Seminavis robusta* and Gene Family Delineation

For expression quantification, nucleotide sequences for the longest isoform of each *S. robusta* gene were retrieved from the recently released *S. robusta* genome assembly (Osuna-Cruz et al., 2020). Next, functional annotations for the *S. robusta* gene models were determined using three different approaches: (i) InterProScan v5.3 (Jones et al., 2014) was used to scan *S. robusta* sequences for matches against the InterPro protein signature databases; (ii) AnnoMine (Vandepoele et al., 2013) was employed to retrieve consensus gene functional annotation from protein similarity searches (using DIAMOND v0.9.9.110; Buchfink et al., 2014), maximum *e*-value 10e − 05) against the Swiss-Prot database (Bairoch and Apweiler, 2000); (iii) eggNOG-mapper (Huerta-Cepas et al., 2017) was executed in DIAMOND mapping mode, based on eggNOG 4.5 orthology data (Huerta-Cepas et al., 2016). All *S. robusta* protein-coding genes together with the genes of 26 eukaryotic genomes were subjected to a DIAMOND all-against-all protein sequence similarity search (max #hits 4000, *e*-value < 10e − 05) to delineate gene families using TribeMCL v10-201.

### RNA Sequencing and Transcriptomic Analysis

A total of 12 sequencing libraries were prepared using an Illumina^®^ TruSeq Stranded mRNA kit. The libraries were sequenced (2 × 75 bp) in one Illumina^®^ NextSeq 500 H150 run. Library preparation and sequencing was performed by VIB Nucleomics Core (VIB, Leuven). The obtained paired-end reads were quality-trimmed by performing a sliding window trimming (settings 4:20) with Trimmomatic (Bolger et al., 2014). Subsequently, the quality of the remaining reads was checked with FastQC^2^. The reads were quasi-mapped to the nucleotide sequences of all *S. robusta* genes (version v1.2, publication in preparation) using Salmon (version 0.9.1) (Patro et al., 2017).

The files containing the transcript quantifications generated by Salmon were imported and aggregated to the gene level using the tximport package (Soneson et al., 2016) for R (version 3.5.1). Differential expression analysis was performed using the R package EdgeR (version 3.22.3) (Robinson et al., 2010; McCarthy et al., 2012). Independent filtering was performed by removing genes with low overall counts [counts-per-million (CPM) < 1 in at least two samples] prior to the analysis (Bourgon et al., 2010). Differences in sequencing depth and RNA population were corrected using a weighted trimmed mean of the log expression ratios (TMM) normalization (Robinson and Oshlack, 2010). Data exploration was performed with a multi-dimensional scaling (MDS) plot visualizing expression differences between samples for the top 500 genes. Negative binomial GLMs, as implemented in the EdgeR software, were used to model read counts for each gene as a function of treatment. For differential expression analysis, three comparisons were defined (DMSO control versus C14-HSL = C vs C14-HSL; DMSO control vs oxo-C14-HSL = C vs oxo-C14-HSL and DMSO control vs TA14 = C vs TA14). Differential expression for each comparison was tested using likelihood ratio tests (LRT) against a log_2_ fold change threshold of 1 (McCarthy and Smyth, 2009), i.e., we require expression to be at least doubled or halved. Based on the fold change threshold test, we consider a gene to be significant if its False Discovery Rate (FDR) adjusted *p*-value is below the 5% level. To derive a list of genes that are significantly differentially expressed (DE) in at least one treatment, we took the union of all significantly DE genes across the three treatments.

## Results

### Growth Response of *Seminavis robusta* to AHL-Homologs and Tetramic Acid

**FIGURE 1:**
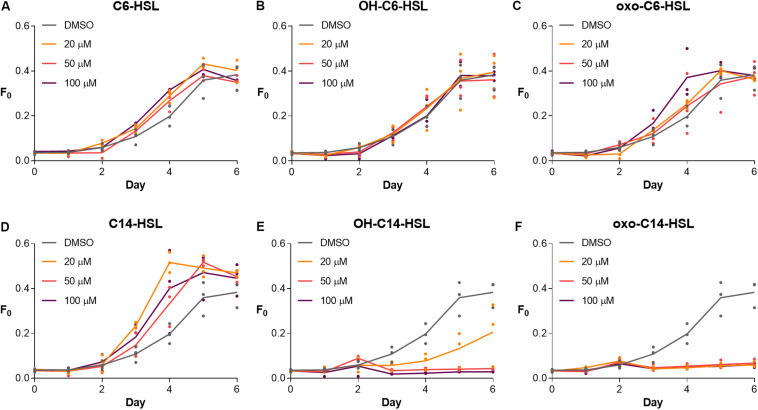
Growth response of *S. robusta* to short- **(A–C)** and long-chained AHLs **(D–F)**. Diatom growth was measured by recording minimum fluorescence (*F*_0_) with a PAM-fluorometer for 6 days. Notably, treatment with C6-HSL, OH-C6-HSL, and oxo-C6-HSL did not induce any changes in growth, whereas C14-HSL treatment stimulated growth in all tested concentrations compared to the DMSO control. In contrast, treatment with OH-C14-HSL and oxo-C14-HSL inhibited growth in all tested concentrations (*n* = 3).

**FIGURE 2:**
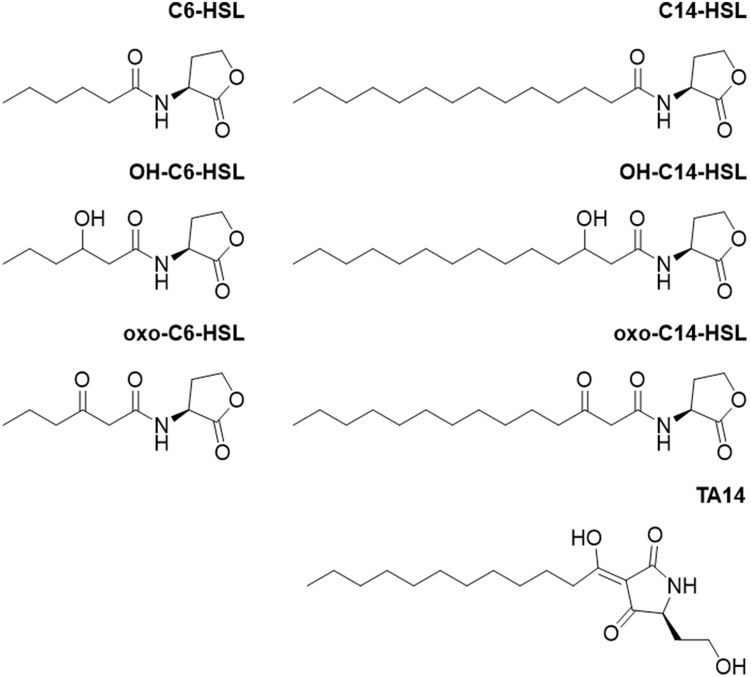
Chemical structures of compounds used in this study. *N*-hexanoyl homoserine lactone = C6-HSL, *N*-3-hydroxyhexanoyl homoserine lactone = OH-C6-HSL, *N*-3-oxohexanoyl homoserine lactone = oxo-C6-HSL, *N*-tetradecanoyl homoserine lactone = C14-HSL, *N*-3-hydroxytetradecanoyl homoserine lactone = OH-C14-HSL, and *N*-3-oxotetradecanoyl homoserine lactone = oxo-C14-HSL.

First, we tested the physiological response of *S. robusta* to short- and long-chained AHLs, including C6-HSL, OH-C6-HSL, oxo-C6-HSL, C14-HSL, OH-C14-HSL, and oxo-C14-HSL with a DMSO treatment serving as control (Figures 1, 2). Cell growth was monitored for a week. Treatment with C6-HSL, OH-C6-HSL, and oxo-C6-HSL did not induce any changes in diatom growth (Figures 1A–C). In contrast, treatment with C14-HSL stimulated diatom growth at all tested concentrations (Figure 1D), while treatment with OH-C14-HSL and oxo-C14-HSL resulted in growth inhibition, especially in case of the latter compound (Figures 1E,F).

Based on these results, an RNA-seq experiment was designed during which *S. robusta* was treated with C14-HSL or oxo-C14-HSL (Figure 2). The growth-inhibiting OH-C14-HSL was not included in the RNA-seq experiment in favor of the oxo-C14-HSL, which exhibits a more pronounced response. In addition, the tetramic acid rearrangement product of oxo-C14-HSL, TA14, was included in the transcriptomics study (Figure 2) as previous work demonstrated that its structural homolog TA12 induces strong growth inhibition in the diatom *Phaeodactylum tricornutum* (Stock F. et al., 2019). During the experiment, cells were counted daily to select a time point for sampling and RNA extraction when differences in growth compared to the control were statistically significant (Supplementary Figure S1). On the third day after addition of C14-HSL, the average cell count had significantly increased, while cell counts had significantly decreased after treatment with oxo-C14-HSL and TA14 (quasi-poisson GLM, *p* < 0.0001; Supplementary Figure S1). The inhibition of growth caused by both oxo-C14-HSL and TA14 is in accordance with previous observations in the pennate diatom *P. tricornutum* (Stock F. et al., 2019). While *N*-3-oxododecanoyl homoserine lactone (oxo-C12-HSL) barely affected growth, its rearrangement product TA12 inhibited growth through a blockage of photosynthetic electron flow (Stock F. et al., 2019). Furthermore, within 24 h 20 μM TA12 was formed from 100 μM oxo-C12-HSL. Assuming that the reaction kinetics of oxo-C14-HSL are similar, it is likely that TA14 had also formed in the treatment with oxo-C14-HSL over the course of three days and caused the observed growth arrest.

### AHL and Tetramic Acid Induced Changes in *S. robusta* Gene Expression

On average, 33.8 million paired-end fragments were obtained per sample. Mapping rates were high for all samples, ranging from 85.8 to 90.2%. In total, 27,359 genes were retained after removing genes with low expression, with a median of 268 paired-end fragments mapped per gene (Supplementary Figure S2).

**FIGURE 3:**
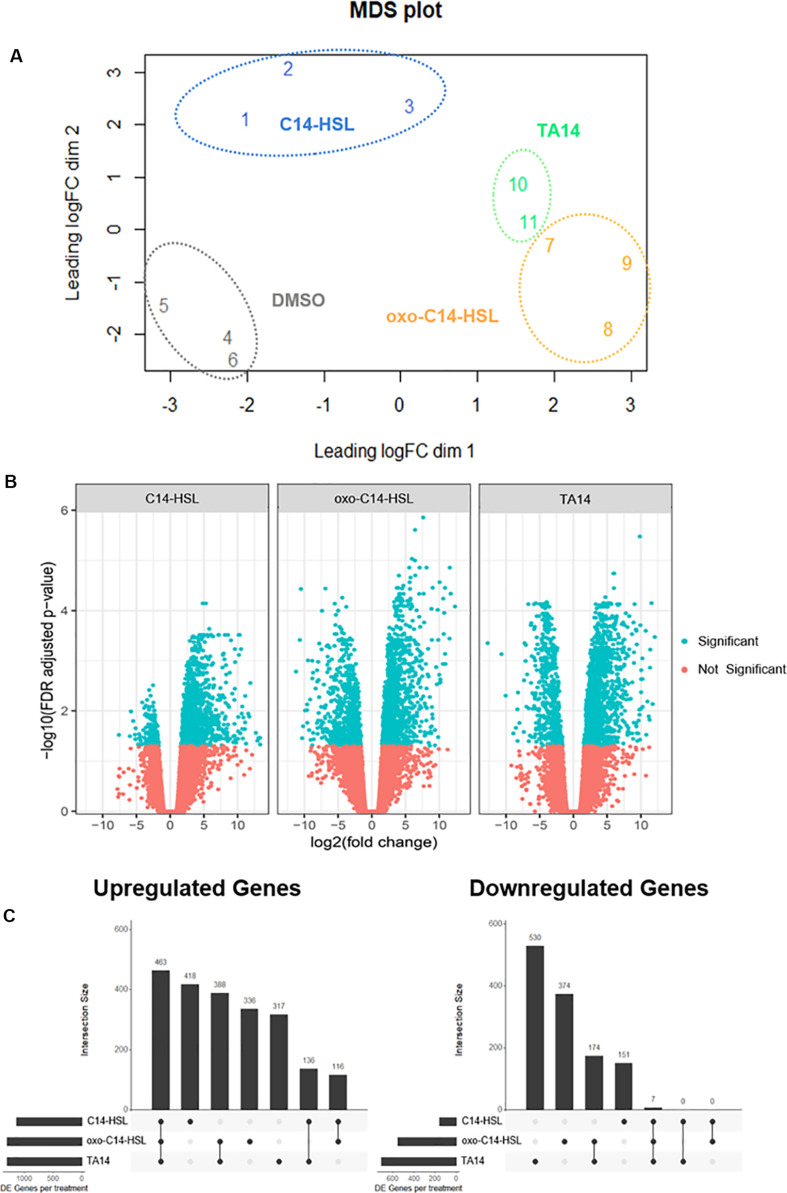
MDS-, Volcano-, and UpSet-plots of transcriptome data. **(A)** MDS plot of RNA-seq data. Distances between samples can be interpreted as the typical log_2_ fold change between them, for the top 500 most discriminative genes. **(B)** Volcano plots of RNA-seq data. Log_2_ fold changes versus the log_10_ FDR adjusted *p*-values for all three treatments when compared to the control. Significantly regulated genes on a 5% FDR level are highlighted in blue. **(C)** UpSet-plots of significantly up- and down-regulated genes in C14-HSL, oxo-C14-HSL, and TA14 treatment (|log_2_ fold change| > 1, FDR ≤ 0.05). The barplot in the top-panel in each plot indicates the number of genes per intersection as depicted in the panel below. Left panel indicates the total number of DE genes per treatment.

RNA-seq data was visualized by MDS based on the expression levels of 500 genes that most discriminated between samples (Ritchie et al., 2015). The plot showed that replicates of each treatment cluster together. This implies that variation between replicates was smaller than variation between treatments. The treatments with oxo-C14-HSL and TA14 had the most similar gene expression profiles, in line with their similar inhibitory effects on growth (Figure 3A). The DMSO control cluster was well separated from all three treatments, suggesting strong differences in expression between the control and all three treatments. For the subsequent differential expression analysis, each treatment was compared to the DMSO control. Out of 27,539 genes, 3,410 were DE in at least one treatment (C14-HSL = 1,291 DE genes; oxo-C14-HSL = 2,015 DE genes; TA14 = 1,858 DE genes). Of these genes, 1,428 are annotated with a molecular function by either InterPro or EggNog.

In total, 2,174 genes (64%) were upregulated in at least one treatment, whereas 1,236 genes (36%) were downregulated. Notably, there was considerably more upregulation than downregulation for all treatments. This was especially pronounced in the presence of C14-HSL (Figures 3B,C). A large fraction of upregulated genes was shared between all treatments (463 genes, 13.5% of all DE genes), whereas only seven downregulated genes (0.2% of all DE genes) were shared (Figure 3C). As already suggested by the MDS analysis, oxo-C14-HSL and TA14 treatments shared more DE genes with each other than they did with C14-HSL. Specifically, oxo-C14-HSL and TA14 shared 388 upregulated genes, compared to 136 and 116 genes shared by oxo-C14-HSL or TA14 and C14-HSL. No overlap in DE genes between C14-HSL and oxo-C14-HSL or TA14 was detected among the downregulated genes.

### C14-HSL, oxo-C14-HSL, and TA14 Induce Signaling Pathways in *Seminavis robusta*

Treatment with C14-HSL, oxo-C14-HSL, and TA14 induced differential expression of 284 genes that are part of the intracellular signaling network (Supplementary Tables S1–S4), the majority of which were upregulated (Supplementary Figure S3).

A total of 129 DE genes contained the InterPro domain “Leucine-rich repeat (LRR) domain superfamily,” 44 of which were described as receptor-like kinases (Supplementary Table S1). In the presence of C14-HSL, genes containing LRR domains were predominantly upregulated (55 genes), while downregulation was only detected in very few cases (six genes). Gene regulation caused by oxo-C14-HSL and TA14 was distributed more evenly, with 24 and 28 upregulated genes, and 27 and 28 downregulated genes, respectively. In addition, we identified differential expression for 15 genes encoding for G-protein coupled receptors (GPCRs) (Supplementary Table S2), all of which contained the InterPro domain “GPCR family 3, GABA-B receptor,” indicating that these proteins share similarities with gamma-aminobutyric acid (GABA)-receptors. GABA receptors are well described in mammals where they primarily bind the neurotransmitters glutamate and GABA, although other members of the GPCR superfamily are known to bind a variety of ligands, e.g., lipids, proteins, and calcium (Port et al., 2013). The conformational change in the receptor induced by binding of the ligand activates the intracellular G-protein that is coupled to the receptor and initiates intracellular signaling processes. Out of the 15 DE GPCR genes, 10 were upregulated in the presence of C14-HSL, compared to seven genes with TA14 and three with oxo-C14-HSL. Only one gene was downregulated, in the treatment with oxo-C14-HSL.

Differential expression was observed for 96 genes with cyclase activity in response to all treatments, e.g., receptor-type guanylate cyclases (45 genes) and members of the nitrilase family (30 genes) (Supplementary Table S3). Guanylate cyclases convert guanosine triphosphate (GTP) into cyclic guanosine monophosphate (cGMP), an important secondary messenger that predominantly activates protein kinases and is thereby involved in several signaling pathways. Almost all DE genes with cyclase activity were upregulated, especially with C14-HSL (60 genes). In the presence of oxo-C14-HSL and TA14, 45 genes were upregulated, while two and five genes were downregulated.

Finally, we detected regulation in 42 genes containing protein kinase domains (Supplementary Table S4), again suggesting a link with secondary messenger signaling. Based on EggNog annotation, these genes included 13 protein kinase kinase kinases (PKKKs) and 12 calcium-dependent kinases. The whole set of genes showed a trend for upregulation, which was again most pronounced in the C14-HSL treatment (31 upregulated genes). In comparison, 19 genes were upregulated with TA14 and 14 genes with oxo-C14-HSL.

### oxo-C14-HSL Treatment Inhibits Expression of Regulatory Cell Cycle Genes

The eukaryotic cell cycle consists of the S-phase, during which DNA is replicated, and the M-phase, where both DNA copies are physically divided. During the cell cycle the S-phase is preceded by Gap-phase 1 (G_1_), while S- and M-phase are separated by Gap-phase 2 (G_2_). Both Gap-phases mark important checkpoints the cell must pass to proceed in its cell cycle (Huysman et al., 2013). Exclusive to the treatment with oxo-C14-HSL, a downregulation of seven regulatory cell cycle genes was observed (Table 1 and Supplementary Table S5). Notably, a cell cycle-specific transcription factor DP1 (Sro1737_g294460), required for the G_1_-S transition of the mitotic cell cycle, was affected (Olson et al., 2010). In addition, we found downregulation of MAD2 (Sro3109_g343920) and BUB1 (Sro690_g187690), two genes required for the mitotic checkpoint, as well as CDC20 (Sro589_g171680), an activation factor for the anaphase promoting complex (APC/C) (Poddar et al., 2005). The APC/C is a protein complex that ubiquitinates mitotic cyclins, resulting in their degradation and leading to a drop in the activity of cyclin-dependent kinases allowing the cell to enter anaphase (Poddar et al., 2005). Moreover, a G_2_-mitotic-specific cyclin (Sro70_g038790) was downregulated.

**TABLE 1:**
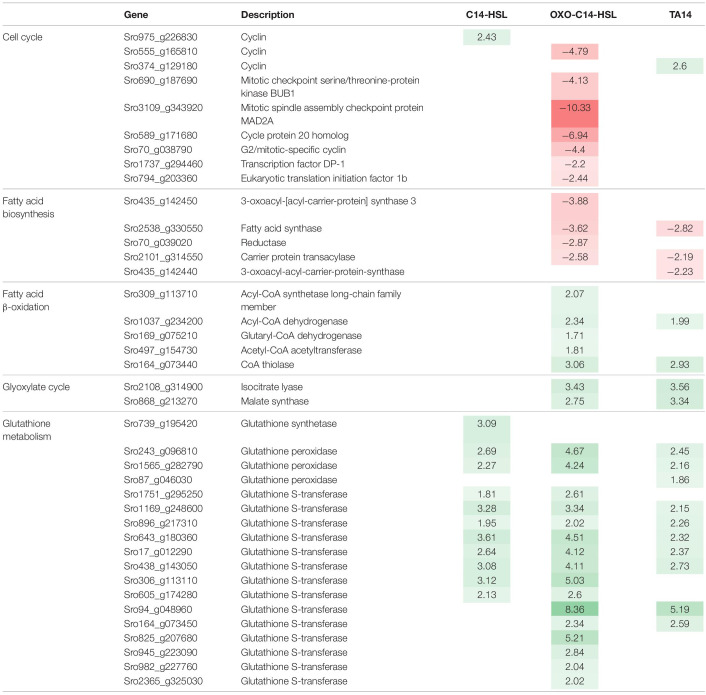
Selection of up- (green) and down-regulated (red) genes relevant to this study.

*Listed genes were involved in cell cycle, fatty acid metabolism, glyoxylate cycle, and glutathione metabolism. Numbers represent log_2_-fold changes and are indicated for those genes which were significantly up- or downregulated (|log_2_| > 1, FDR < 0.05).*

The strong downregulation of cell cycle genes by oxo-C14-HSL pointed towards cell cycle arrest most likely at the transition from G_1_ to S-phase. Indeed, cell growth in the oxo-C14-HSL treatment stagnated during the second day of culturing (Supplementary Figure S1).

### oxo-C14-HSL and TA14 Induce a Shift in the Lipid Metabolism of *Seminavis robusta*

**FIGURE 4:**
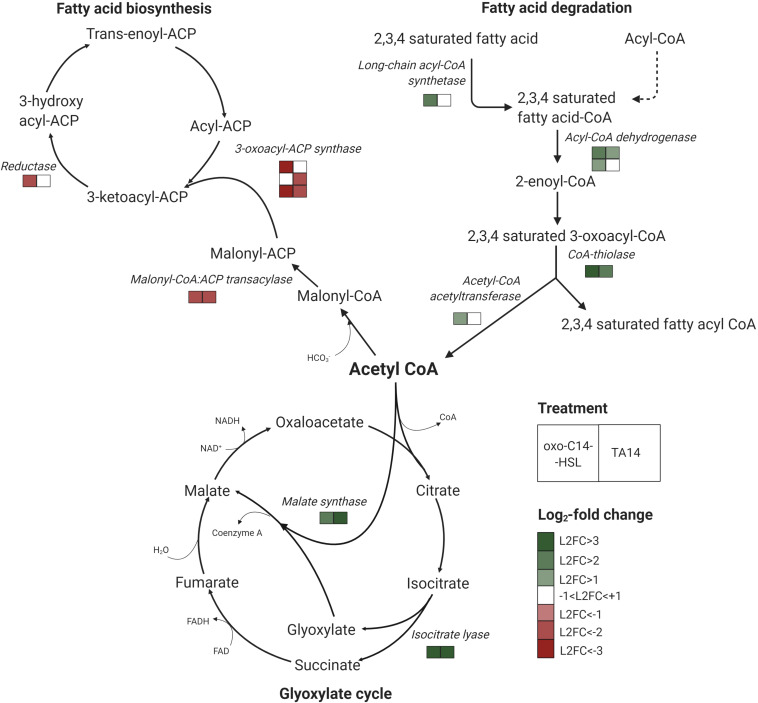
Transcriptomic response of lipid metabolism and glyoxylate cycle genes in *S. robusta* after oxo-C14-HSL (left square) and TA14 treatment (right square). Multiple rows of squares indicate the number of enzymes that were identified with significant differential expression in *S. robusta*. Legend shows log_2_-fold changes (L2FC) of treatment vs DMSO control. Graph was created with BioRender.

Treatments with oxo-C14-HSL and TA14 induced marked transcriptional changes in fatty acid biosynthesis and degradation, which were not observed with C14-HSL (Figure 4, Table 1, and Supplementary Table S6). Five genes involved in fatty acid biosynthesis were downregulated, specifically a carrier protein transacylase (Sro2101_g314550), a fatty acid synthase (Sro2538_g330550), two 3-oxoacyl-[acyl-carrier-protein] synthases (Sro435_g142450 and Sro435_g142440) and an oxidoreductase (Sro70_g039020). In contrast, five genes encoding for enzymes involved in the degradation of fatty acids by β-oxidation were upregulated (Figure 4 and Supplementary Table S6), these being a long chain acyl-CoA synthetase (Sro309_g113710), which initiates β-oxidation, an acyl-CoA dehydrogenase (Sro1037_g234200), a glutaryl-CoA dehydrogenase (Sro169_g075210), a CoA thiolase (Sro164_g073440) and an acetyl-CoA acetyltransferase (Sro497_g154730). Together, these results suggest that oxo-C14-HSL and TA14 induce a shift in lipid metabolism towards increased fatty acid degradation.

### oxo-C14-HSL and TA14 Induce Upregulation of Two Key Enzymes of the Glyoxylate Cycle

The glyoxylate cycle shares five out of eight enzymes with the mitochondrial tricarboxylic acid cycle (TCA) and can process acetyl-CoA generated during β-oxidation into malate, which can subsequently be transported into mitochondria (Smith et al., 2016). In contrast to the TCA cycle, the glyoxylate cycle is located in peroxisomes. It comprises two key enzymes: isocitrate lyase which forms glyoxylate and succinate from isocitrate, and malate synthase which catalyzes the production of malate from glyoxylate and acetyl-CoA (Figure 4). Each enzyme is encoded by only one gene in *S. robusta* (Sro2108_g314900 and Sro868_g213270, respectively). In the oxo-C14-HSL and TA14 treatments both genes were upregulated, while no differential expression was induced by C14-HSL (Figure 4, Table 1, and Supplementary Table S7). Excess oxaloacetate generated in this pathway can be further processed into storage carbohydrates by gluconeogenesis, which is supported by the upregulation of two fructose-1,6-bisphosphatases (Sro1842_g301030 and Sro1029_g233230) in the oxo-C14-HSL and TA14 treatments (Supplementary Table S7).

### Photosynthesis Is Affected by oxo-C14-HSL and TA14

In the presence of oxo-C14-HSL and TA14, differential expression of genes encoding 20 light harvesting proteins was observed, all of which contain a chlorophyll *a*/*b* binding domain (Supplementary Table S8). In contrast, none of these genes were affected by C14-HSL. Using gene family prediction, it was found that 17 of the 20 DE light harvesting genes belonged to two gene families (Supplementary Table S8). Interestingly, genes belonging to the light harvesting complex (LHC) LHCF gene family were almost exclusively downregulated, and comprise genes encoding LHCF proteins, the main light harvesting proteins in PSI and PSII. In contrast, genes of the other gene family, which are closely related to an *lhcx* gene in *P. tricornutum*, were exclusively upregulated. LHCX proteins mechanistically disconnect the LHC antennae from PSII, thereby dissipating excess light energy as heat to prevent oxidative damage of the photosystems by reactive oxygen species (ROS) (Buck et al., 2019). This data suggests that photoprotection mechanisms were upregulated in *S. robusta* while photosynthesis was downregulated in response to oxo-C14-HSL and TA14.

**FIGURE 5:**
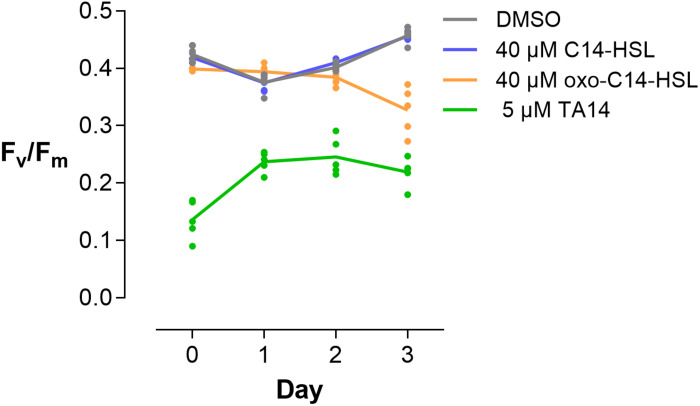
Photosynthetic efficiency of *S. robusta*. *F*_*v*_/*F*_*m*_ of *S. robusta* was measured over 3 days of treatment with DMSO, 40 μM C14-HSL, 40 μM oxo-C14-HSL, and 5 μM TA14. Measurements were taken with the same cell culture flasks in which cells were cultured for RNA extraction. Lines connect means of five individually plotted replicates.

This change in gene expression fits to the physiological measurements that were done prior to RNA extraction. Exposure to C14-HSL did not affect photosynthetic efficiency (*F*_*v*_/*F*_*m*_), nor did it induce differential expression in light harvesting proteins (Figure 5). In contrast, *F*_*v*_/*F*_*m*_ decreased in the presence of oxo-C14-HSL over the course of 3 days, which substantiates the observation that this treatment downregulated LHCF coding genes and upregulated *lhcx*-like genes. TA14 addition, however, had an immediate negative effect on photosynthetic efficiency (Figure 5, see data on day 0) from which cells seemed to recover, if only marginally. This sharp decrease in *F*_*v*_/*F*_*m*_ suggests that the photosystems of *S. robusta* were immediately damaged by TA14 addition, similar to what has been observed when TA12 was added to *P. tricornutum* cultures (Stock F. et al., 2019).

### AHL and TA Treatment Induces a Detoxification-Like Response in *Seminavis robusta*

A collection of 18 genes involved in glutathione metabolism, including one glutathione synthetase, three glutathione peroxidases and 14 glutathione *S*-transferases were also upregulated (Table 1 and Supplementary Table S9). Glutathione is a metabolite with multiple functions but is most commonly regarded as a potent antioxidant. It is involved in the detoxification of ROS, hence the upregulation of glutathione related genes is often seen as an indicator for oxidative stress. Glutathione synthetases produce glutathione from glycine, while glutathione peroxidases oxidize glutathione to glutathione disulfide and glutathione *S*-transferases attach glutathione via an S-bond to various substrates for detoxification. Overall, the most genes (16 genes) were upregulated in the presence of oxo-C14-HSL, followed by 11 and 10 genes with C14-HSL and TA14, respectively.

## Discussion

Quorum sensing compounds released by bacteria do not only play an important role as signaling compounds among conspecific cells but are also documented to be involved in interkingdom signaling between bacteria and eukaryotes. In algae, for example, exposure to AHLs has been shown to cause chemokinesis or trigger mechanisms that either lead to AHL degradation or production of AHL-mimics (Teplitski et al., 2004; Joint et al., 2007; Syrpas et al., 2014). However, molecular studies on the response of microalgae to AHLs are lacking.

In this study, we show that short-chained AHLs do not seem to have an obvious effect on the growth of the marine benthic diatom *S. robusta*. In contrast, the long-chained C14-HSL results in growth stimulation while OH-C14-HSL, oxo-C14-HSL, and the rearrangement product tetramic acid inhibit its growth (Figures 1D–F and Supplementary Figure S1). Arguably, the applied concentrations were relatively high (40 μM C14-HSL and OH-C14-HSL and 5 μM TA14). However, AHL concentrations within the diffusion-limited micro-environment of biofilms may accumulate to high levels as suggested by the report of >3,000 μM *N*-dodecanoyl homoserine lactone in a subtidal biofilm (Huang et al., 2007). A subsequent RNA-seq experiment revealed strong transcriptional changes underlying these very distinct growth responses. Overall, the expression profiles in response to oxo-C14-HSL and TA14 were more similar to each other compared to the C14-HSL exposure, which fits to the growth data. Considering expected kinetics of the transformation of AHLs into tetramic acids in seawater (Stock F. et al., 2019), it can be estimated that 40 μM oxo-C14-HSL could potentially produce 8 μM TA14 within 1 day. This suggests that tetramic acid had formed in the treatment with oxo-C14-HSL and hence that gene expression was likely also affected by the accumulation of its TA rearrangement product.

A common response among all treatments was the marked upregulation of numerous genes involved in intracellular signaling, including genes encoding cyclases, protein kinases, GPCRs and proteins with LRR-domains. The highest number of upregulated genes potentially involved in intracellular signaling was observed in the treatment with growth-stimulating C14-HSL. Currently, little is known about intracellular signaling cascades in diatoms, but most of the identified genes in this study are similar to genes involved in intracellular signaling in plants (Hughes and Sperandio, 2008). Proteins with LRR domains, for instance, can function as receptors on the cell surface or in the cytoplasm, and are crucial to relay information about biotic and abiotic stimuli through a downstream signaling cascade (Yue et al., 2012). The signaling cascade also includes calcium-dependent kinases (Ligterink and Hirt, 2001) that eventually modulate the plant’s response to an external stimulus like bacteria, e.g., by upregulating its defense system or by inducing changes in its cell cycle. A high number of DE genes involved in intracellular signaling was also observed in a previous study investigating transcriptional and metabolic changes in a co-culture system of the diatom *Thalassiosira pseudonana* and the vitamin B_12_-producing bacterium *Ruegeria pomeroyi* (Durham et al., 2017). Similarly, Cirri et al. (2019) noted an upregulation of a high number of guanylate cyclases after exposing *S. robusta* to spent medium of the bacteria *Maribacter* sp. and *Roseovarius* sp. despite their opposite effects on the sexual reproduction of the diatom.

In addition to the modulation of intracellular signaling in response to all tested compounds, we detected distinct changes in gene expression dependent on the AHL or TA applied. The downregulation of seven genes encoding regulatory cell cycle proteins fits well with the growth-inhibiting effect of oxo-C14-HSL (Supplementary Figure S1). Similarly, a study on the diatom *Thalassiosira weissflogii* reported cell cycle arrest in the G_1_-S phase after treatment with the aldehyde decadienal, which was eventually followed by cell death (Casotti et al., 2005). It is conceivable that downregulation of cell cycle genes in the oxo-C14-HSL treatment was also a precursor for cell death, especially when considering the likely accumulation of TA14 during the course of the experiment (see above). Moreover, it was shown that TA14 is more toxic compared to TA12 which induced cell death in *P. tricornutum* at a concentration of 20 μM (Stock F. et al., 2019). Interestingly, we observed that fatty acid biosynthesis was downregulated in the growth-inhibiting oxo-C14-HSL and TA14 treatments, while fatty acid degradation was upregulated. Durham et al. (2017) also identified downregulation of twelve genes involved in fatty acid biosynthesis when *T. pseudonana* was grown in co-culture with *R. pomeroyi*, although co-culture with the bacterium stimulated algal growth in this study. At this point it can only be speculated what this shift in lipid metabolism in *S. robusta* could indicate. Possibly, *S. robusta* used the supplied AHL as an external energy source. Since AHLs have long acyl-side chains their metabolization would likely involve fatty acid degradation. However, the use of AHLs as an energy source does not explain why fatty acid degradation was unaffected in the growth-stimulating C14-HSL treatment. Further studies are required to elucidate the putative metabolization of AHLs in diatom cultures, e.g., by adding hydroxyl- or keto-forms of C14 fatty acids to diatom cultures. Intriguingly, we also detected upregulation of key enzymes of the glyoxylate cycle with oxo-C14-HSL and TA14, suggesting that lipid metabolism is somehow linked to the glyoxylate cycle (Figure 4). During germination of plant seeds, the glyoxylate cycle was observed to metabolize lipids into carbohydrates to generate an energy source for germination (Gonzalez and Vodkin, 2007). However, whether a similar link is operational in diatoms remains unclear. Alternatively, upregulation of fatty acid degradation could also point toward the production of defense molecules, e.g., unsaturated aldehydes and jasmonic acid. In diatoms, one of the most common defense strategies is the production of unsaturated aldehydes such as decadienal, which is synthesized from arachidonic acid (Pohnert, 2002). In a study on *Thalassiosira rotula* they demonstrated that phospholipase A2 initiates the reaction cascade by cleaving fatty acid residues from phospholipids (Pohnert, 2002). Even though mechanical stress is hypothesized to be crucial for the activation of phospholipase A2, we detected upregulation of this enzyme (Sro1901_g304330) in the presence of oxo-C14-HSL and TA14 (Supplementary Table S10). This finding is also in line with a previous study on *S. robusta*, where an increase in arachidonic acid was detected after treatment with bacterial spent medium (Cirri et al., 2019). In contrast to unsaturated aldehydes, jasmonic acid is formed from the C18 precursor linolenic acid, which is transformed into 12-oxo-phytodienoic acid (OPDA) and is subsequently converted into jasmonic acid by OPDA reductase (OPR3) and three rounds of β-oxidation (Koo, 2017). Cirri et al. (2019) also described upregulation of an oxophytodienoate reductase-like (OPR) protein (Sro250_g098890) in *S. robusta* when exposed to bacterial spent medium. Interestingly, we also identified strong upregulation of the same gene in all three treatments (Supplementary Table S10). Together with the upregulation of β-oxidation, this could also be an indicator for jasmonic acid production in the oxo-C14-HSL and TA14 treatments.

Exposure to oxo-C14-HSL and TA14 resulted in a decrease in photosynthetic efficiency and induced downregulation of genes coding for light-harvesting LHCF proteins, whereas LHCX proteins that are involved in photoprotection were upregulated. The concomitant upregulation of glutathione related genes, especially glutathione *S*-transferases, suggests that this disturbance of photosynthesis contributed to the build-up of ROS and the activation of detoxification by glutathione. However, we also observed upregulation of glutathione-related genes in the C14-HSL treatment which was likely caused by other factors.

In conclusion, we show that *S. robusta* responds via distinct and unique gene regulation upon treatment with AHL analogs and tetramic acid. Treatment with the growth-stimulating C14-HSL led to upregulation of many genes involved in intracellular signaling. In contrast, growth-inhibiting oxo-C14-HSL and its tetramic acid rearrangement product TA14 predominantly induced stress-related responses, including a shift in lipid metabolism towards increased fatty acid degradation and downregulation of cell cycle genes. In benthic biofilms, *S. robusta* is likely to be exposed to a wide range of AHLs and concentrations can vary considerably at small spatial scales. Combining our results with those of Cirri et al. (2019) provides a general overview of the transcriptomic response to the presence of bacteria and their AHLs. A list of 20 marker genes that were DE in all treatments in the present study, as well as in response to treatment with spent medium of *Maribacter* sp. and *Roseobacter* sp. in Cirri et al. (2019) is provided in Supplementary Table S11. Interestingly, these marker genes contain three thioredoxin-like, a peroxiredoxin-like and a glutathione *S*-transferase gene, indicating that the induction of antioxidant production in response to AHLs seems to be part of a more general response to bacterial signals. Moreover, among the marker genes we find three short-chain dehydrogenase/reductase (SDR) enzymes, two alcohol dehydrogenases, two methyltransferases and an OPR-like protein. Unraveling their function and regulation is a prime target for future research on bacteria-diatom interactions. In addition, a better understanding of the temporal- and concentration-dependent dynamics of the diatom’s response to bacterial signaling compounds is needed. This could provide a better documentation of the role of AHLs as a chemical cue for diatoms to sense and distinguish between bacteria in their surroundings.

Our study provides a first glimpse into the complex network of chemical interactions in marine biofilms, where diatoms and bacteria play important roles as primary producers and in biogeochemical cycling (Underwood and Kromkamp, 1999). Studying diatom-bacteria interactions in these biofilms is complicated by their small-scale heterogeneity and the complexity of these assemblages (Jesus et al., 2005). The application of next-generation sequencing techniques allows researchers to get mechanistic insights into the nature and strength of these interactions. Based on our results, we propose that AHL signaling can play a key role in shaping biofilm communities, either by signaling the presence of potential harmful or advantageous bacteria to the diatom, or as an energy source. Metagenomic and metatranscriptomic approaches, possibly combined with the use of labeled AHLs, are needed to investigate these interactions in natural biofilms in addition to laboratory studies.

## Data Availability Statement

The data for this study have been deposited in the European Nucleotide Archive (ENA) at EMBL-EBI under accession number PRJEB35041 (https://www.ebi.ac.uk/ena/data/view/PRJEB35041).

## Author Contributions

WV, SM, and FS designed the study. FS executed the wet-lab experiments for this study, and wrote the manuscript and all other authors contributed with proofreading and critical feedback. FS performed the computational analyses with help from GB and SD. KoV developed the scripts. CO-C, EV, LD, and KlV provided the *Seminavis* reference genome and functional annotation. SM provided the compounds used in this study.

## Conflict of Interest

The authors declare that the research was conducted in the absence of any commercial or financial relationships that could be construed as a potential conflict of interest.

## Footnotes

1 http://bccm.belspo.be

2 http://www.bioinformatics.babraham.ac.uk/projects/fastqc
