# Supplementary material for: Distinctive Growth and Transcriptional Changes of the Diatom Seminavis robusta in Response to Quorum Sensing Related Compounds
Source: Front Microbiol. 2020 Jun 9;11:1240. doi: 10.3389/fmicb.2020.01240 (PMC7296067; doi:10.3389/fmicb.2020.01240)
Supplement: Supplementary file 1 [file Data_Sheet_1.docx]

Supplementary Material

# Supplementary Figures


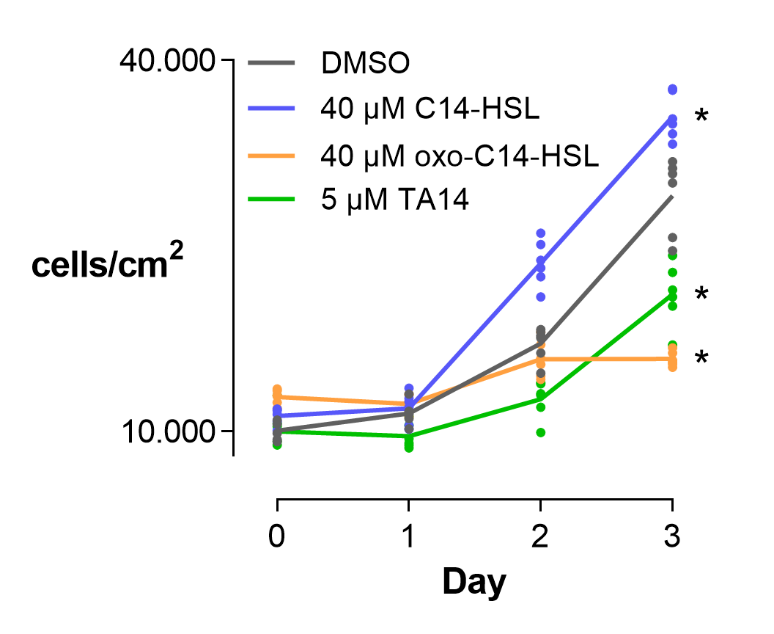


**Supplementary Figure 1**. Growth response of *S. robusta* prior to RNA extraction. Cell counts of *S. robusta* during treatment with C14-HSL, oxo-C14-HSL and TA14 and a DMSO control over three days. Lines connect means of five individually plotted replicates. On the third day, cell counts were significantly increased (C14-HSL) or decreased (oxo-C14-HSL and TA14) compared to the DMSO control, indicated with “*” (tested with quasi-Poisson GLM where all treatments were compared to the DMSO control). This time point was chosen for RNA extraction.


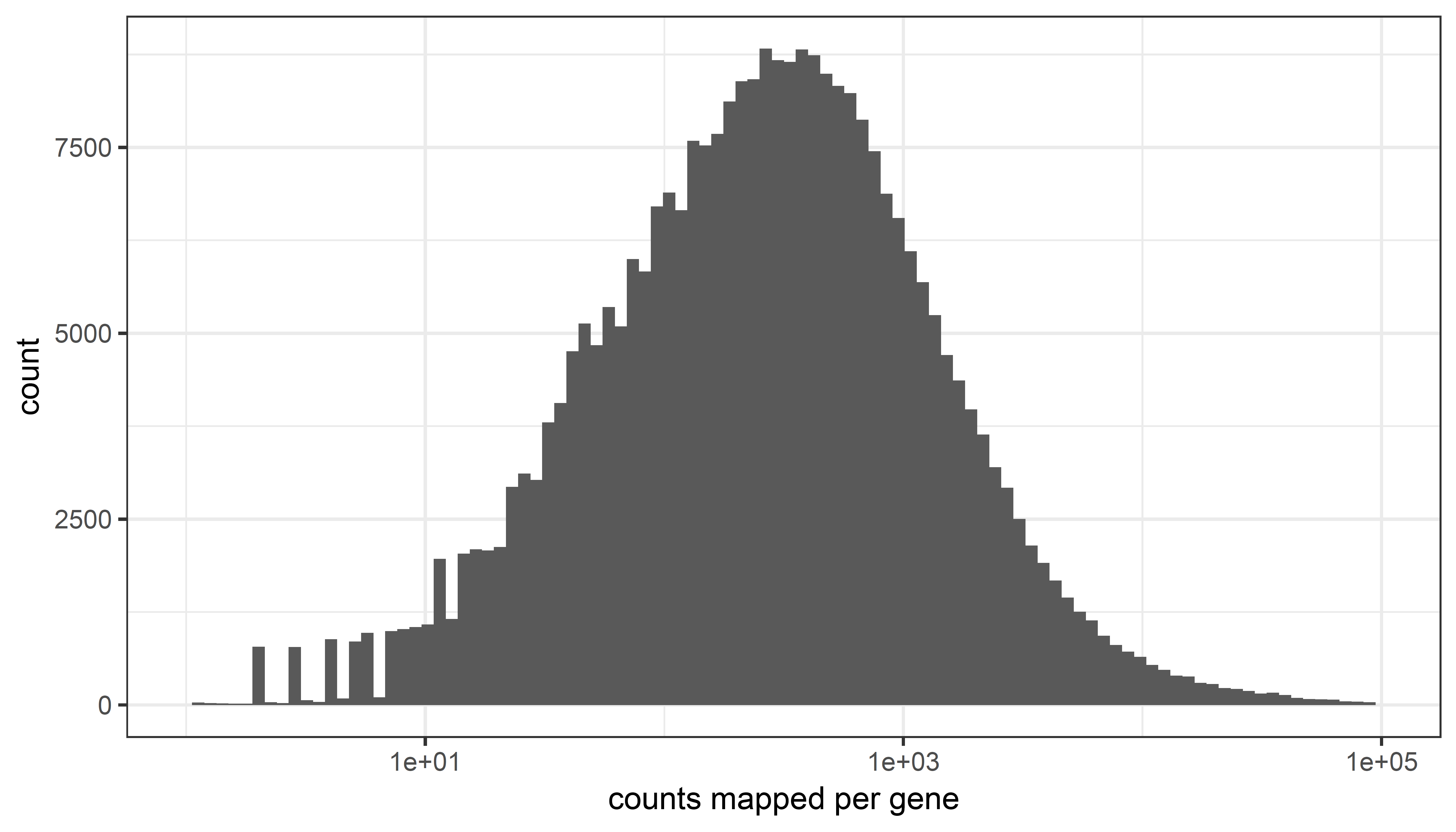


**Supplementary Figure 2.** Distribution of read counts per gene. Median = 268 reads.


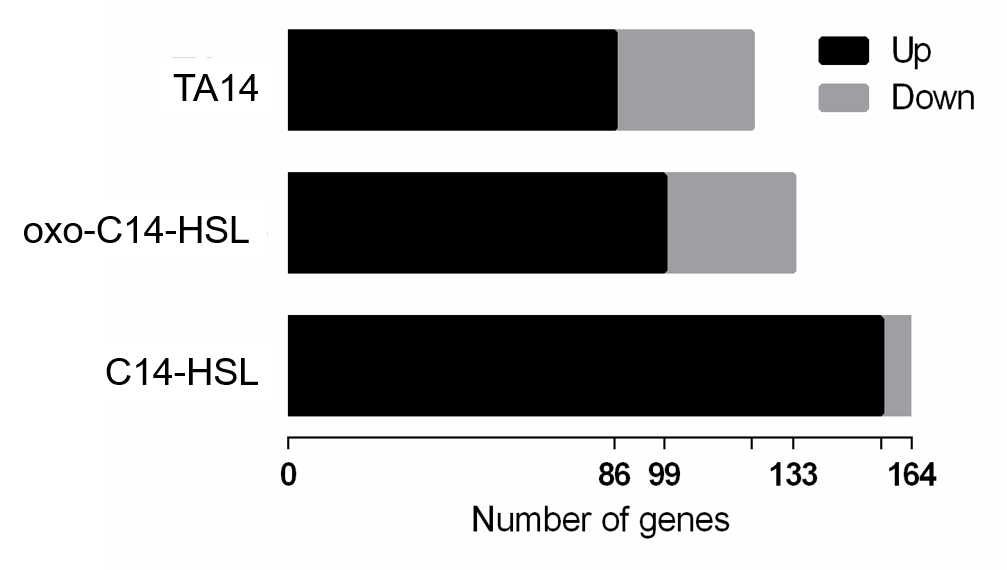


**Supplementary Figure 3.** Distribution of up-and downregulated genes related to “signaling” in all three treatments.

# Supplementary Tables

**Supplementary Table 1**. Differentially expressed proteins with LRR domain.

| **Gene_ID** | **C v. C14-HSL** | **C v. oxo-C14-HSL** | **C v. TA14** |
| --- | --- | --- | --- |
| Sro45_g026870 | 2.02 | NA | NA |
| Sro24_g016630 | 1.97 | NA | NA |
| Sro3306_g346490 | NA | NA | 6.99 |
| Sro343_g122030 | NA | NA | 2.39 |
| Sro269_g104020 | 4.69 | 4.39 | NA |
| Sro274_g105540 | -1.92 | NA | NA |
| Sro107_g053920 | 4.72 | NA | NA |
| Sro380_g130700 | 3.38 | NA | NA |
| Sro139_g065150 | NA | -2.83 | NA |
| Sro606_g174530 | 3.62 | NA | NA |
| Sro1468_g275160 | -5.80 | NA | NA |
| Sro1024_g232720 | NA | -3.42 | NA |
| Sro52_g031030 | 2.35 | NA | NA |
| Sro425_g140240 | NA | -1.71 | -2.62 |
| Sro155_g070580 | NA | 3.65 | 4.57 |
| Sro409_g137240 | NA | -1.73 | -2.99 |
| Sro307_g113420 | NA | -1.97 | NA |
| Sro106_g053470 | NA | 2.21 | NA |
| Sro1707_g292650 | NA | NA | -2.83 |
| Sro213_g088310 | NA | -4.62 | NA |
| Sro409_g137230 | 3.02 | NA | NA |
| Sro853_g211190 | 6.24 | NA | NA |
| Sro122_g059180 | NA | NA | -3.21 |
| Sro995_g229140 | 2.51 | NA | NA |
| Sro692_g188080 | 3.93 | 3.45 | NA |
| Sro1048_g235200 | NA | NA | -2.03 |
| Sro449_g145300 | 3.73 | NA | NA |
| Sro105_g053250 | -1.88 | NA | NA |
| Sro1644_g288160 | 5.01 | 3.50 | 5.23 |
| Sro905_g218560 | NA | 3.18 | 4.06 |
| Sro32_g021020 | 2.64 | NA | NA |
| Sro291_g109440 | 3.15 | NA | NA |
| Sro2940_g340670 | NA | NA | 3.07 |
| Sro1150_g246700 | 3.22 | NA | NA |
| Sro1114_g242810 | -2.64 | NA | NA |
| Sro32_g020790 | 3.07 | NA | 2.14 |
| Sro5_g003960 | 4.48 | 3.12 | NA |
| Sro32_g020780 | 4.08 | 2.31 | 3.39 |
| Sro503_g155840 | NA | 3.17 | 2.81 |
| Sro52_g031200 | 4.69 | NA | NA |
| Sro1343_g264670 | 6.60 | NA | NA |
| Sro2004_g310430 | NA | NA | -5.55 |
| Sro2829_g338080 | 6.16 | NA | NA |
| Sro2_g001280 | NA | NA | -4.55 |
| Sro594_g172440 | NA | NA | 9.48 |
| Sro52_g031210 | 9.36 | NA | NA |
| Sro127_g061000 | 2.72 | NA | NA |
| Sro979_g227240 | NA | -2.24 | -2.25 |
| Sro830_g208230 | 3.35 | NA | NA |
| Sro490_g153530 | NA | -4.80 | NA |
| Sro1171_g248850 | -2.18 | NA | NA |
| Sro570_g168590 | 2.62 | NA | NA |
| Sro544_g163621 | 2.02 | NA | NA |
| Sro522_g159560 | NA | 3.18 | 4.08 |
| Sro649_g181170 | NA | 2.39 | 2.57 |
| Sro265_g102970 | NA | -2.70 | NA |
| Sro1128_g244291 | -2.54 | NA | NA |
| Sro1928_g306040 | 4.91 | NA | NA |
| Sro1104_g241810 | NA | NA | -2.80 |
| Sro1369_g266960 | NA | NA | -5.06 |
| Sro1113_g242630 | 3.30 | NA | NA |
| Sro496_g154670 | 4.11 | 4.71 | NA |
| Sro1545_g281350 | NA | NA | 2.43 |
| Sro820_g207300 | 2.16 | NA | 2.86 |
| Sro278_g106650 | 2.69 | NA | NA |
| Sro338_g120850 | NA | -8.11 | NA |
| Sro305_g112750 | NA | -5.78 | NA |
| Sro7_g005830 | 3.67 | NA | NA |
| Sro7_g005840 | 5.02 | NA | NA |
| Sro7_g005860 | 4.12 | NA | NA |
| Sro7_g005850 | 5.01 | NA | NA |
| Sro127_g060870 | NA | NA | 2.47 |
| Sro840_g209400 | 3.94 | 4.27 | NA |
| Sro566_g167830 | NA | NA | -4.02 |
| Sro548_g164440 | 2.64 | NA | NA |
| Sro862_g212490 | NA | NA | -3.04 |
| Sro1550_g281700 | NA | -2.76 | -2.48 |
| Sro566_g167840 | NA | NA | -4.14 |
| Sro737_g195170 | 2.72 | 2.54 | 3.13 |
| Sro502_g155650 | NA | -2.66 | NA |
| Sro489_g153270 | NA | -2.88 | NA |
| Sro213_g088580 | NA | -3.50 | NA |
| Sro638_g179570 | NA | -2.46 | -3.04 |
| Sro2225_g319770 | 6.69 | NA | NA |
| Sro2239_g320230 | 4.51 | NA | NA |
| Sro390_g132860 | NA | NA | 1.92 |
| Sro729_g193880 | NA | 2.48 | 3.32 |
| Sro41_g025320 | NA | -1.93 | -4.01 |
| Sro2_g001190 | NA | 5.25 | 6.18 |
| Sro38_g023750 | 4.05 | 2.76 | 2.81 |
| Sro38_g023760 | 5.24 | 3.74 | 4.10 |
| Sro38_g023740 | 3.52 | NA | NA |
| Sro753_g197280 | 4.41 | NA | NA |
| Sro533_g161600 | NA | NA | -2.91 |
| Sro984_g227880 | NA | NA | -4.55 |
| Sro1131_g244630 | NA | NA | -3.75 |
| Sro1293_g260150 | NA | 2.88 | NA |
| Sro170_g075340 | 5.64 | 4.09 | 5.03 |
| Sro1544_g281240 | 2.30 | 1.92 | 2.68 |
| Sro537_g162330 | NA | -6.36 | -6.28 |
| Sro3424_g347870 | 2.79 | NA | NA |
| Sro305_g112700 | NA | -3.07 | -2.97 |
| Sro2502_g329520 | NA | NA | -6.61 |
| Sro560_g166690 | NA | 2.43 | NA |
| Sro219_g090270 | NA | 2.29 | NA |
| Sro602_g173770 | NA | -3.49 | NA |
| Sro1788_g297580 | NA | -4.06 | NA |
| Sro1788_g297610 | NA | -7.95 | NA |
| Sro1213_g252970 | NA | NA | -2.87 |
| Sro284_g107970 | NA | NA | -5.73 |
| Sro2414_g326820 | 3.40 | 2.57 | 2.49 |
| Sro733_g194530 | 2.16 | NA | NA |
| Sro2522_g330200 | 9.56 | NA | 9.39 |
| Sro1080_g238990 | 7.58 | NA | 6.62 |
| Sro125_g060160 | NA | -4.78 | NA |
| Sro398_g134730 | NA | NA | 2.59 |
| Sro1639_g287820 | NA | -1.93 | -2.10 |
| Sro1364_g266460 | NA | NA | -2.94 |
| Sro1282_g259031 | NA | NA | 4.75 |
| Sro116_g057040 | 1.74 | NA | NA |
| Sro1253_g256380 | 1.95 | NA | NA |
| Sro436_g142680 | NA | NA | -4.05 |
| Sro433_g141910 | NA | -2.29 | NA |
| Sro2450_g328060 | NA | NA | 2.00 |
| Sro45_g027200 | NA | NA | 2.46 |
| Sro1777_g296950 | NA | -2.96 | NA |
| Sro1579_g283760 | NA | -3.25 | -2.73 |
| Sro370_g128470 | NA | -2.14 | NA |
| Sro5_g003950 | 4.20 | NA | NA |

**Supplementary Table 2**. Differentially expressed G-proteins coupled receptors.

| **Gene_ID** | **Description** | **C v. C14-HSL** | **C v. oxo-C14-HSL** | **C v. TA14** |
| --- | --- | --- | --- | --- |
| Sro618_g176190 | Gamma-aminobutyric acid (GABA) B receptor | 8.03 | 6.14 | 6.95 |
| Sro1778_g296990 |  | 3.88 | NA | 3.72 |
| Sro164_g073460 |  | 2.98 | NA | 3.07 |
| Sro206_g086510 |  | 2.64 | NA | NA |
| Sro1124_g243790 |  | 2.46 | NA | NA |
| Sro1272_g258200 |  | 3.37 | NA | NA |
| Sro346_g122700 |  | 3.33 | NA | NA |
| Sro1803_g298650 |  | NA | -3.11 | NA |
| Sro1566_g282880 |  | NA | 4.79 | 4.99 |
| Sro107_g053750 |  | 2.81 | NA | NA |
| Sro38_g023860 |  | NA | NA | 2.15 |
| Sro787_g202420 |  | NA | NA | 2.11 |
| Sro3052_g342840 |  | NA | 1.89 | NA |
| Sro708_g190670 |  | 4.93 | NA | NA |
| Sro183_g079720 |  | 3.57 | NA | 3.63 |

**Supplementary Table 3.** Differentially expressed genes with cyclase activity.

| **Gene** | **Description** | **C v. C14-HSL** | **C v. oxo-C14-HSL** | **C v. TA14** |
| --- | --- | --- | --- | --- |
| Sro2252_g320860 | Receptor-type guanylate cyclase gcy | 3.73 | 3.99 | 3.58 |
| Sro938_g222300 | Receptor-type guanylate cyclase gcy | 3.01 | 3.12 | 3.94 |
| Sro1084_g239470 | Receptor-type guanylate cyclase gcy | 3.84 | 2.75 | 3.27 |
| Sro27_g018150 | Receptor-type guanylate cyclase gcy | 5.49 | 5.49 | NA |
| Sro1658_g289110 | Guanylate cyclase | 4.88 | 3.30 | NA |
| Sro149_g068410 | Receptor-type guanylate cyclase gcy | NA | 4.40 | 4.25 |
| Sro76_g041670 | Receptor-type guanylate cyclase gcy | 4.06 | NA | NA |
| Sro76_g041710 | Receptor-type guanylate cyclase gcy | 3.15 | NA | NA |
| Sro198_g083980 | Receptor-type guanylate cyclase gcy | 2.91 | NA | NA |
| Sro662_g183400 | Receptor-type guanylate cyclase gcy | 2.23 | NA | NA |
| Sro1240_g255340 | Receptor-type guanylate cyclase gcy | 1.98 | NA | NA |
| Sro467_g148960 | Receptor-type guanylate cyclase gcy | NA | 3.03 | NA |
| Sro2765_g336630 | Receptor-type guanylate cyclase gcy | NA | 2.76 | NA |
| Sro1266_g257560 | Receptor-type guanylate cyclase gcy | NA | 2.75 | NA |
| Sro882_g215340 | Receptor-type guanylate cyclase gcy | NA | 2.28 | NA |
| Sro893_g217020 | natriuretic peptide receptor | 7.46 | 5.47 | 5.22 |
| Sro67_g037780 | Receptor-type guanylate cyclase gcy | 7.03 | 4.71 | 5.76 |
| Sro1817_g299530 | natriuretic peptide receptor 2 | 4.34 | 4.46 | 4.37 |
| Sro1520_g279370 | Receptor-type guanylate cyclase gcy | 4.44 | 4.65 | NA |
| Sro181_g079010 | Receptor-type guanylate cyclase gcy | 4.36 | 4.60 | NA |
| Sro354_g124710 | - | 4.34 | 3.84 | NA |
| Sro3162_g344640 | Receptor-type guanylate cyclase gcy | 6.52 | NA | 6.17 |
| Sro34_g022090 | Receptor-type guanylate cyclase gcy | NA | 6.25 | 6.61 |
| Sro109_g054500 | Receptor-type guanylate cyclase gcy | NA | 4.68 | 3.92 |
| Sro471_g149630 | Receptor-type guanylate cyclase gcy | NA | 3.78 | 4.62 |
| Sro997_g229450 | - | NA | 3.47 | 3.31 |
| Sro420_g139400 | Receptor-type guanylate cyclase gcy | NA | 3.35 | 3.30 |
| Sro553_g165400 | Receptor-type guanylate cyclase gcy | NA | 3.51 | 3.07 |
| Sro953_g224220 | Receptor-type guanylate cyclase gcy | NA | 2.41 | 3.27 |
| Sro98_g050660 | Receptor-type guanylate cyclase gcy | NA | 1.88 | 1.87 |
| Sro898_g217570 | Receptor-type guanylate cyclase gcy | 7.48 | NA | NA |
| Sro1_g000890 | Receptor-type guanylate cyclase gcy | 6.79 | NA | NA |
| Sro1086_g239700 | Receptor-type guanylate cyclase gcy | 4.82 | NA | NA |
| Sro873_g213980 | Receptor-type guanylate cyclase gcy | 4.73 | NA | NA |
| Sro1775_g296850 | Receptor-type guanylate cyclase gcy | 3.89 | NA | NA |
| Sro366_g127640 | Receptor-type guanylate cyclase gcy | 3.81 | NA | NA |
| Sro2031_g311830 | Ephrin type-B receptor 3 (Fragment) | 3.76 | NA | NA |
| Sro1800_g298430 | Receptor-type guanylate cyclase gcy | 3.58 | NA | NA |
| Sro222_g091250 | Receptor-type guanylate cyclase gcy | 3.37 | NA | NA |
| Sro602_g173750 | Receptor-type guanylate cyclase gcy | 3.17 | NA | NA |
| Sro1615_g286180 | Receptor-type guanylate cyclase gcy | 2.97 | NA | NA |
| Sro156_g070930 | Guanylate cyclase | 2.94 | NA | NA |
| Sro2972_g341210 | Receptor-type guanylate cyclase gcy | 2.72 | NA | NA |
| Sro100_g051350 | Receptor-type guanylate cyclase gcy | 2.68 | NA | NA |
| Sro1447_g273590 | Receptor-type guanylate cyclase gcy | 2.56 | NA | NA |
| Sro310_g114030 | Receptor-type guanylate cyclase gcy | 2.55 | NA | NA |
| Sro34_g022100 | Receptor-type guanylate cyclase gcy | 2.50 | NA | NA |
| Sro6_g005510 | - | 2.34 | NA | NA |
| Sro2139_g316150 | Receptor-type guanylate cyclase gcy | 2.17 | NA | NA |
| Sro886_g216200 | Receptor-type guanylate cyclase gcy | 2.04 | NA | NA |
| Sro43_g026230 | Receptor-type guanylate cyclase gcy | 1.86 | NA | NA |
| Sro509_g157020 | Receptor-type guanylate cyclase gcy | NA | 7.64 | NA |
| Sro15_g011420 | - | NA | -5.30 | NA |
| Sro18_g012740 | cyclase soluble subunit alpha-1 | NA | NA | 2.73 |
| Sro9_g007450 | - | NA | NA | 2.17 |
| Sro647_g180890 | Receptor-type guanylate cyclase gcy | NA | NA | 2.17 |
| Sro790_g202800 | - | NA | NA | -3.23 |
| Sro2443_g327830 | - | NA | NA | -4.54 |
| Sro550_g164700 | Nitrilase family, member 2 | 7.18 | NA | 6.78 |
| Sro1213_g252950 | Nitrilase family, member 2 | NA | NA | 9.66 |
| Sro743_g196100 | Nitrilase family, member 2 | 3.07 | 2.73 | 4.34 |
| Sro97_g050100 | Nitrilase family, member 2 | 1.94 | 2.09 | 2.46 |
| Sro710_g190980 | Nitrilase family, member 2 | 4.75 | NA | NA |
| Sro132_g062490 | Nitrilase family, member 2 | NA | NA | 6.38 |
| Sro888_g216470 | Nitrilase family, member 2 | NA | NA | 3.58 |
| Sro1279_g258830 | Nitrilase family, member 2 | NA | NA | 3.37 |
| Sro1321_g262500 | - | NA | NA | -2.43 |
| Sro167_g074370 | - | NA | NA | -2.91 |
| Sro97_g050060 | - | 8.28 | 8.90 | 9.93 |
| Sro886_g216190 | Nitrilase family, member 2 | 5.98 | 8.01 | 10.72 |
| Sro658_g182810 | Nitrilase family, member 2 | 5.37 | 6.80 | 6.79 |
| Sro5_g004230 | Nitrilase family, member 2 | 5.53 | 5.54 | 6.48 |
| Sro149_g068300 | Nitrilase family, member 2 | 3.93 | 6.04 | 6.58 |
| Sro475_g150430 | Nitrilase family, member 2 | 7.44 | 5.11 | 4.01 |
| Sro5_g004210 | Nitrilase family, member 2 | 5.90 | 4.83 | 5.58 |
| Sro23_g015620 | Nitrilase family, member 2 | 5.43 | 4.51 | 5.03 |
| Sro97_g050080 | - | 4.45 | 3.97 | 4.01 |
| Sro258_g101140 | Nitrilase family, member 2 | 3.21 | 2.58 | 3.06 |
| Sro2_g001270 | Nitrilase family, member 2 | 2.60 | 2.41 | 3.34 |
| Sro61_g034940 | Nitrilase family, member 2 | 2.79 | 2.14 | 2.80 |
| Sro703_g190090 | Nitrilase family, member 2 | 5.93 | 6.13 | NA |
| Sro97_g050130 | Nitrilase family, member 2 | NA | 10.10 | 11.36 |
| Sro5_g004220 | Nitrilase family, member 2 | NA | 4.68 | 6.43 |
| Sro1024_g232660 | Nitrilase family, member 2 | NA | 3.21 | 4.59 |
| Sro717_g191980 | Nitrilase family, member 2 | NA | 2.65 | 2.84 |
| Sro270_g104340 | Nitrilase family, member 2 | 6.21 | NA | NA |
| Sro2749_g336210 | Nitrilase family, member 2 | 2.79 | NA | NA |
| Sro97_g050050 | Nitrilase family, member 2 | 2.39 | NA | NA |
| Sro68_g038050 | Nitrilase family, member 2 | NA | 8.39 | NA |
| Sro100_g051260 | - | NA | -2.87 | NA |
| Sro2086_g313860 | Nitrilase family, member 2 | NA | NA | 11.29 |
| Sro17_g012190 | Nitrilase family, member 2 | NA | NA | 4.54 |
| Sro2243_g320450 | Nitrilase family, member 2 | NA | NA | 3.20 |
| Sro1321_g262510 | - | NA | NA | -5.30 |
| Sro608_g174890 | activated protein kinase catalytic subunit alpha-1 | 2.84 | NA | NA |
| Sro2792_g337210 | activated protein kinase catalytic subunit alpha-1 | 2.75 | NA | NA |

**ST 4**. Differentially expressed proteins with kinase domain.

| **Gene_ID** | **Description** | **C v. C14-HSL** | **C v. oxo-C14-HSL** | **C v. TA14** |
| --- | --- | --- | --- | --- |
| Sro258_g101100 | calcium-dependent protein kinase | 2.70 | NA | 2.23 |
| Sro32_g020770 | serine threonine-protein kinase | 3.62 | 2.69 | 2.67 |
| Sro138_g064770 | S_TKc | 2.30 | 2.23 | 2.75 |
| Sro139_g065130 | calcium-dependent protein kinase | 3.13 | NA | 3.22 |
| Sro138_g064760 | calcium-dependent protein kinase | 2.23 | 2.37 | 3.28 |
| Sro198_g084080 | calcium-dependent protein kinase | 2.30 | 2.42 | 3.66 |
| Sro338_g120890 | calcium-dependent protein kinase | 3.80 | 3.79 | 5.29 |
| Sro1497_g277590 | calcium-dependent protein kinase | 5.84 | 5.88 | 7.21 |
| Sro90_g047490 | Lipopolysaccharide kinase (Kdo/WaaP) family | -2.43 | NA | NA |
| Sro1013_g231310 | calcium-dependent protein kinase | 2.20 | NA | NA |
| Sro1430_g271930 | calcium-dependent protein kinase | 3.68 | NA | NA |
| Sro372_g128880 | calcium-dependent protein kinase | 3.14 | NA | NA |
| Sro68_g038070 | calcium-dependent protein kinase | 2.41 | NA | NA |
| Sro634_g178990 | calcium-dependent protein kinase | NA | -2.34 | NA |
| Sro337_g120630 | calcium-dependent protein kinase | 3.06 | NA | NA |
| Sro790_g202800 | Guanylate cyclase | NA | NA | -3.23 |
| Sro9_g007450 | #NV | NA | NA | 2.17 |
| Sro1086_g239700 | Guanylate cyclase | 4.82 | NA | NA |
| Sro15_g011420 | Guanylate cyclase | NA | -5.30 | NA |
| Sro366_g127640 | Guanylate cyclase | 3.81 | NA | NA |
| Sro886_g216200 | Guanylate cyclase | 2.04 | NA | NA |
| Sro2031_g311830 | Guanylate cyclase | 3.76 | NA | NA |
| Sro452_g145870 | AarF domain containing kinase | 2.88 | 3.91 | 2.99 |
| Sro298_g111200 | AarF domain containing kinase | 6.29 | 3.97 | 3.79 |
| Sro601_g173600 | protein kinase kinase kinase | 2.85 | NA | 2.31 |
| Sro448_g145210 | protein kinase kinase kinase | NA | NA | 2.34 |
| Sro106_g053590 | protein kinase kinase kinase | 2.78 | 1.97 | 2.64 |
| Sro252_g099590 | protein kinase kinase kinase | 3.54 | 1.96 | 2.79 |
| Sro2811_g337610 | protein kinase kinase kinase | 3.02 | 2.76 | 3.20 |
| Sro816_g206710 | protein kinase kinase kinase | NA | 2.13 | 3.25 |
| Sro183_g079780 | protein kinase kinase kinase | 3.71 | 3.65 | 3.64 |
| Sro119_g057960 | protein kinase kinase kinase | 4.29 | 3.41 | 4.75 |
| Sro421_g139540 | protein kinase kinase kinase | 4.32 | NA | NA |
| Sro91_g047560 | protein kinase kinase kinase | 3.91 | NA | NA |
| Sro119_g058090 | protein kinase kinase kinase | NA | -1.80 | NA |
| Sro608_g174890 | Guanylate cyclase | 2.84 | NA | NA |
| Sro57_g033520 | protein kinase kinase kinase | NA | -2.33 | NA |
| Sro3560_g349180 | protein kinase kinase kinase | -2.80 | NA | NA |
| Sro690_g187690 | budding uninhibited by benzimidazoles 1 homolog, beta | NA | -4.13 | NA |
| Sro2562_g331340 | 0 | 2.18 | NA | NA |
| Sro2792_g337210 | Guanylate cyclase | 2.75 | NA | NA |
| Sro412_g137930 | 0 | 1.99 | NA | NA |

**Supplementary Table 5.** Differentially expressed cell cycle genes.

| **Gene_ID** | **Description** | **C v. C14-HSL** | **C v. oxo-C14-HSL** | **C v. TA14** |
| --- | --- | --- | --- | --- |
| Sro975_g226830 | G1/S-specific cyclin-E1 | 2.43 | NA | NA |
| Sro555_g165810 | complex that (...) regulates the cell-cycle during G(1) S transition | NA | -4.79 | NA |
| Sro374_g129180 | complex that (...) regulates the cell-cycle during G(1) S transition | NA | NA | 2.60 |
| Sro690_g187690 | Mitotic checkpoint serine/threonine-protein kinase BUB1 | NA | -4.13 | NA |
| Sro3109_g343920 | Mitotic spindle assembly checkpoint protein MAD2A | NA | -10.33 | NA |
| Sro589_g171680 | cycle protein 20 homolog | NA | -6.94 | NA |
| Sro70_g038790 | G2/mitotic-specific cyclin | NA | -4.40 | NA |
| Sro1737_g294460 | Transcription factor DP-1 | NA | -2.20 | NA |
| Sro794_g203360 | Eukaryotic translation initiation factor 1b | NA | -2.44 | NA |

**Supplementary Table 6.** Differential expression of genes involved in fatty acid biosynthesis and degradation.

| **Process** | **Gene_ID** | **Description** | **C v. C14-HSL** | **C v. oxo-C14-HSL** | **C v. TA14** |
| --- | --- | --- | --- | --- | --- |
| **Fatty acid biosynthesis** | Sro435_g142450 | 3-oxoacyl-[acyl-carrier-protein] synthase 3 | NA | -3.88 | NA |
|  | Sro2538_g330550 | fatty acid synthase | NA | -3.62 | -2.82 |
|  | Sro70_g039020 | reductase | NA | -2.87 | NA |
|  | Sro2101_g314550 | carrier protein transacylase | NA | -2.58 | -2.19 |
|  | Sro435_g142440 | 3-oxoacyl-[acyl-carrier-protein] synthase 1 | NA | NA | -2.23 |
| **Fatty acid ß-oxidation** | Sro309_g113710 | Acyl-CoA synthetase long-chain family member | NA | 2.07 | NA |
|  | Sro1037_g234200 | acyl-CoA dehydrogenase | NA | 2.34 | 1.99 |
|  | Sro169_g075210 | glutaryl-CoA dehydrogenase | NA | 1.71 | NA |
|  | Sro497_g154730 | Acetyl-CoA acetyltransferase | NA | 1.81 | NA |
|  | Sro164_g073440 | CoA thiolase | NA | 3.06 | 2.93 |

**Supplementary Table 7.** Differentially expressed genes of the Glyoxylate cycle and Glucolysis/ Gluconeogenesis.

| **Process** | **Gene_ID** | **Description** | **C v. C14-HSL** | **C v. oxo-C14-HSL** | **C v. TA14** |
| --- | --- | --- | --- | --- | --- |
| **Glyoxylate cycle** | Sro2108_g314900 | Isocitrate lyase | NA | 3.43 | 3.56 |
|  | Sro868_g213270 | malate synthase | NA | 2.75 | 3.34 |
| **Glucolysis**  **& Gluconeo-genesis** | Sro15_g011300 | Glyceraldehyde-3-phosphate dehydrogenase (Fragment) | NA | -2.70 | -2.96 |
|  | Sro664_g183640 | phosphoglycerate mutase | NA | NA | -2.31 |
|  | Sro867_g213140 | 3-bisphosphoglycerate-dependent phosphoglycerate mutase | -2.01 | NA | NA |
|  | Sro1842_g301030 | Fructose-1,6-bisphosphatase | NA | 2.99 | 1.93 |
|  | Sro1029_g233230 | Fructose-1,6-bisphosphatase | NA | 2.53 | 2.28 |

**Supplementary Table 8.** Differentially expressed genes associated with light harvesting.

| **Gene_ID** | **Gene family** | **Description** | **C v. C14-HSL** | **C v. oxo-C14-HSL** | **C v. TA14** |
| --- | --- | --- | --- | --- | --- |
| Sro86_g045670 | HOM02SEM000043 | Fucoxanthin-chlorophyll a-c binding protein | NA | 3.51 | NA |
| Sro533_g161680 |  | Fucoxanthin-chlorophyll a-c binding protein | NA | -4.02 | -3.28 |
| Sro1451_g273830 |  | Fucoxanthin-chlorophyll a-c binding protein | NA | -2.05 | NA |
| Sro236_g095070 |  | Fucoxanthin chlorophyll a c | NA | -1.85 | NA |
| Sro1043_g234820 |  | Fucoxanthin-chlorophyll a-c binding protein | NA | -2.52 | -2.34 |
| Sro285_g108140 |  | Fucoxanthin-chlorophyll a-c binding protein | NA | -2.56 | NA |
| Sro25_g016900 |  | Fucoxanthin-chlorophyll a-c binding protein | NA | -2.03 | NA |
| Sro6_g005380 |  | Fucoxanthin-chlorophyll a-c binding protein | NA | -2.25 | NA |
| Sro63_g035900 |  | Fucoxanthin-chlorophyll a-c binding protein | NA | -2.02 | NA |
| Sro31_g020150 |  | Fucoxanthin-chlorophyll a-c binding protein A, chloroplastic | NA | NA | -1.86 |
| Sro149_g068340 | HOM02SEM000080 | b binding protein L1818, chloroplastic | NA | 2.51 | NA |
| Sro844_g209920 |  | Fucoxanthin chlorophyll a c | NA | NA | 9.79 |
| Sro1207_g252450 |  | b binding protein L1818, chloroplastic | NA | NA | 5.21 |
| Sro652_g181650 |  | Fucoxanthin chlorophyll a c | NA | 3.77 | 4.82 |
| Sro412_g137900 |  | Fucoxanthin chlorophyll a c | NA | 4.10 | 4.59 |
| Sro829_g208070 |  | Fucoxanthin chlorophyll a c | NA | 3.89 | NA |
| Sro288_g108750 |  | b binding protein L1818, chloroplastic | NA | 2.31 | NA |
| Sro74_g040880 | HOM02SEM000096 | Chlorophyll A-B binding protein | NA | 3.94 | 3.82 |
| Sro132_g062570 | HOM02SEM000096 | Chlorophyll A-B binding protein | NA | 3.63 | NA |
| Sro851_g210830 | HOM02SEM000395 | Fucoxanthin chlorophyll a c | NA | -2.25 | NA |

**Supplementary Table 9.** Differentially expressed genes involved in glutathione metabolism.

| **Gene_ID** | **Description** | **C v. C14-HSL** | **C v. oxo-C14-HSL** | **C v. TA14** |
| --- | --- | --- | --- | --- |
| Sro1751_g295250 | glutathione Stransferase | 1.81 | 2.61 | NA |
| Sro243_g096810 | glutathione peroxidase | 2.69 | 4.67 | 2.45 |
| Sro1565_g282790 | glutathione peroxidase | 2.27 | 4.24 | 2.16 |
| Sro87_g046030 | glutathione peroxidase | NA | NA | 1.86 |
| Sro739_g195420 | glutathione synthetase | 3.09 | NA | NA |
| Sro1169_g248600 | microsomal glutathione S-transferase | 3.28 | 3.34 | 2.15 |
| Sro896_g217310 | Glutathione S-transferase | 1.95 | 2.02 | 2.26 |
| Sro945_g223090 | Glutathione S-transferase | NA | 2.84 | NA |
| Sro643_g180360 | Microsomal glutathione S-transferase | 3.61 | 4.51 | 2.32 |
| Sro17_g012290 | Glutathione S-Transferase | 2.64 | 4.12 | 2.37 |
| Sro438_g143050 | 0 | 3.08 | 4.11 | 2.73 |
| Sro164_g073450 | Glutathione S-Transferase | NA | 2.34 | 2.59 |
| Sro825_g207680 | glutathione Stransferase | NA | 5.21 | NA |
| Sro94_g048960 | Glutathione S-transferase, N-terminal domain | NA | 8.36 | 5.19 |
| Sro306_g113110 | Glutathione S-transferase, N-terminal domain | 3.12 | 5.03 | NA |
| Sro605_g174280 | glutathione Stransferase | 2.13 | 2.60 | NA |
| Sro982_g227760 | Glutathione S-Transferase | NA | 2.04 | NA |
| Sro2365_g325030 | Glutathione S-Transferase | NA | 2.02 | NA |

**Supplementary Table 10.** Differentially expressed genes involved in biosynthesis of defense molecules.

| **Gene_ID** | **Description** | **C v. C14-HSL** | **C v. oxo-C14-HSL** | **C v. TA14** |
| --- | --- | --- | --- | --- |
| Sro1901_g304330 | Phospholipase A2, group XIIA | NA | 2.54 | 2.04 |
| Sro250_g098890 | 12-oxophytodienoate reductase | 4.35 | 5.61 | 4.92 |

**Supplementary Table 11**. Top 20 differentially expressed marker genes of *S. robusta* responding to bacterial exudates and bacterial signaling molecules.

| **Gene_ID** | **Description** | **C v. C14-HSL** | **C v. oxo-C14-HSL** | **C v. TA14** | ***Maribacter* exudates** | ***Roseovarius* exudates** |
| --- | --- | --- | --- | --- | --- | --- |
| Sro250_g098890 | oxophytodienoate reductase-like protein | 4.35 | 5.58 | 4.94 | 7.26 | 6.11 |
| Sro481_g151640 | Short-chain dehydrogenase/reductase | 10.51 | 12.32 | 7.24 | 5.22 | 4.90 |
| Sro1926_g305900 | Short-chain dehydrogenase/reductase | 3.75 | 5.52 | 2.86 | 11.11 | 8.82 |
| Sro660_g183080 | Short-chain dehydrogenase/reductase | 2.31 | 3.45 | 3.08 | 5.89 | 4.48 |
| Sro989_g228490 | Alcohol dehydrogenase | 1.86 | 3.92 | 2.79 | 6.08 | 4.11 |
| Sro48_g028090 | Hydroxysteroid alcohol dehydrogenase | 2.92 | 3.99 | 3.12 | 4.51 | 3.08 |
| Sro1947_g307130 | Methyltransferase | 2.89 | 3.16 | 1.93 | 5.09 | 4.16 |
| Sro1693_g291670 | Methyltransferase | 5.61 | 5.70 | 4.23 | 5.73 | 4.76 |
| Sro728_g193680 | NAD(P)-binding dehydratase | 8.54 | 10.84 | 6.78 | 3.07 | 3.30 |
| Sro701_g189830 | Thioredoxin-like | 3.43 | 3.35 | 2.77 | 7.00 | 5.50 |
| Sro420_g139380 | Thioredoxin-like | 3.37 | 4.21 | 3.84 | 4.52 | 3.48 |
| Sro194_g082670 | Thioredoxin | 4.57 | 5.49 | 3.14 | 3.33 | 2.57 |
| Sro923_g220770 | Peroxiredoxin-like | 4.61 | 3.59 | 4.06 | 2.83 | 2.65 |
| Sro643_g180360 | Putative Glutathione S-transferase | 3.60 | 4.48 | 2.34 | 4.80 | 3.17 |
| Sro558_g166350 | Dispatched homolog | 3.25 | 3.22 | 2.01 | 5.93 | 5.53 |
| Sro2066_g313280 | Unknown DUF1330 | 4.52 | 8.79 | 3.28 | 9.38 | 5.72 |
| Sro44_g026730 | Unknown | 9.58 | 10.72 | 7.58 | 5.92 | 4.41 |
| Sro876_g214460 | Unknown | 10.15 | 11.68 | 5.80 | 6.13 | 4.03 |
| Sro886_g216180 | Unknown | 4.57 | 8.10 | 12.08 | 6.67 | 4.37 |
| Sro747_g196590 | Tetratricopeptide-like helical domain | 3.74 | 5.89 | 7.57 | 2.92 | 2.53 |
